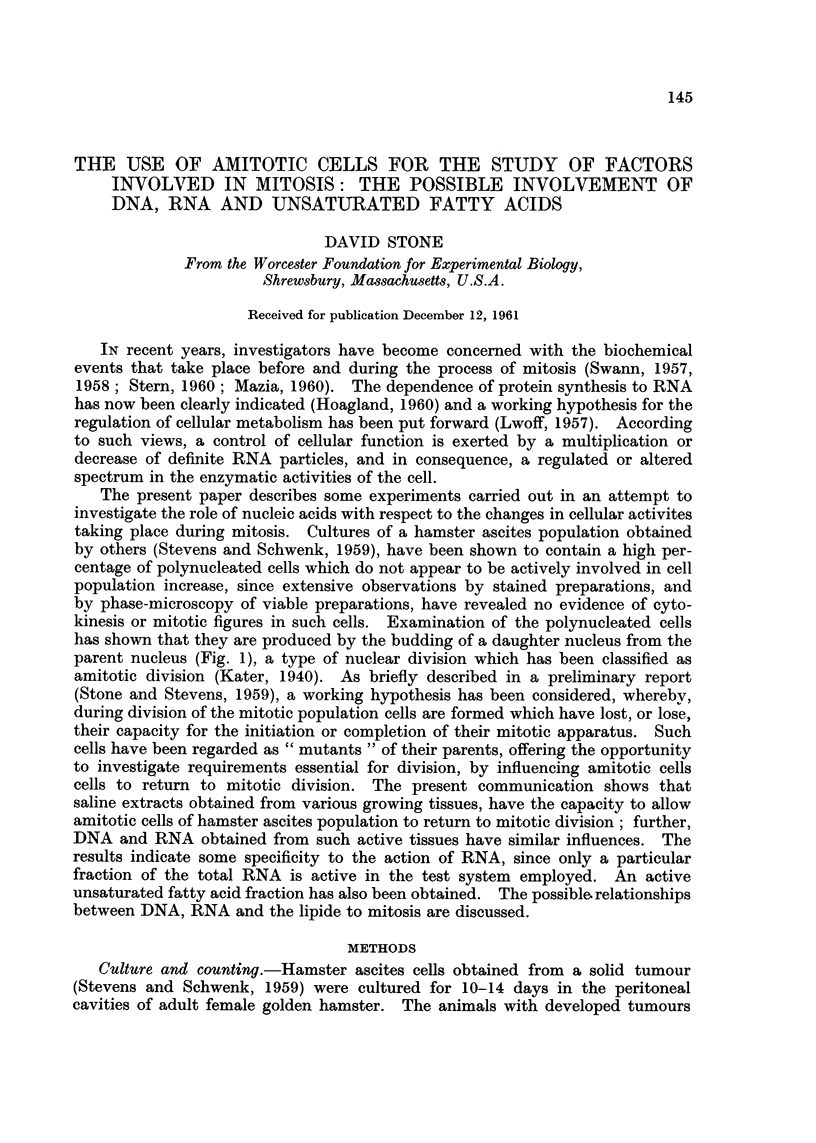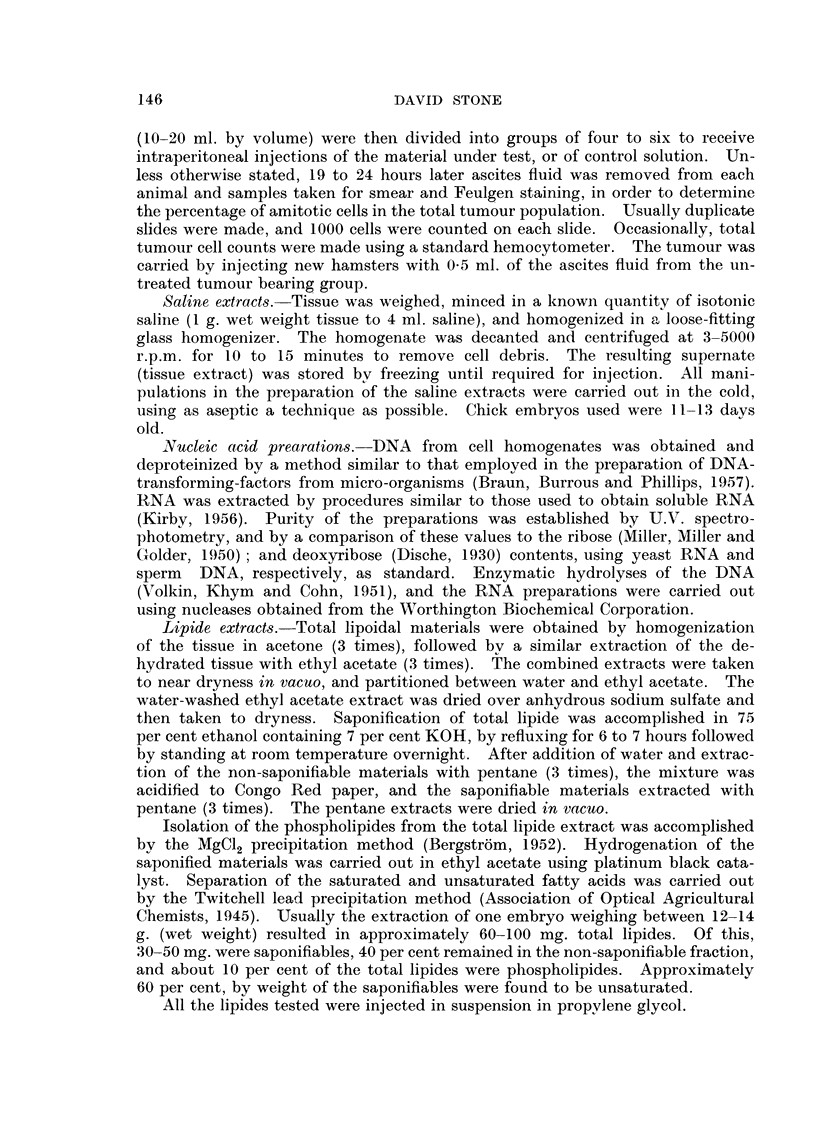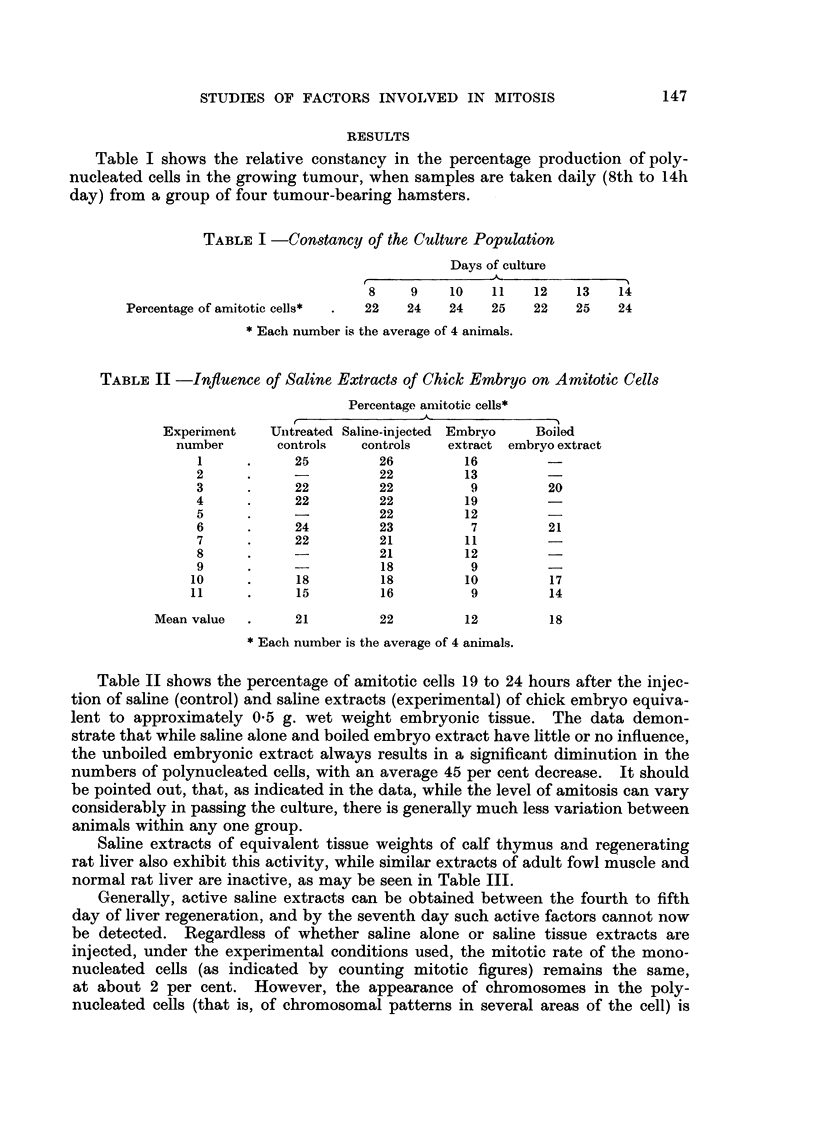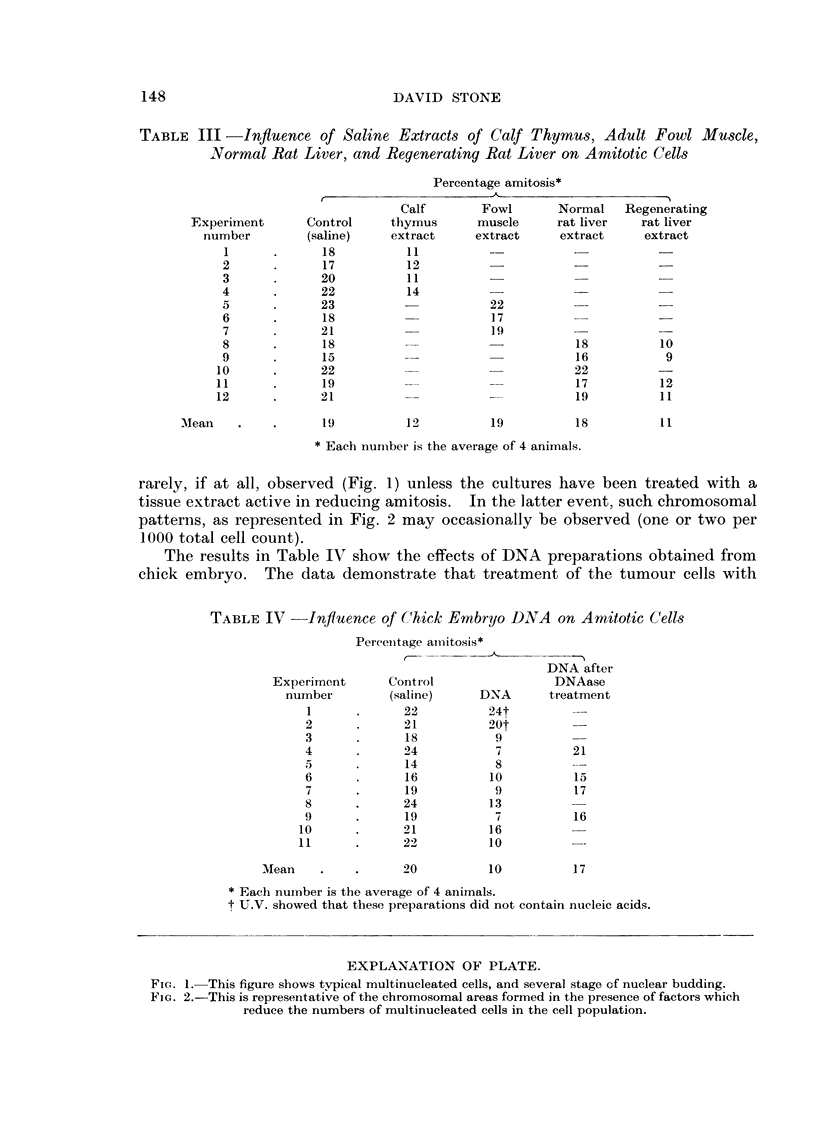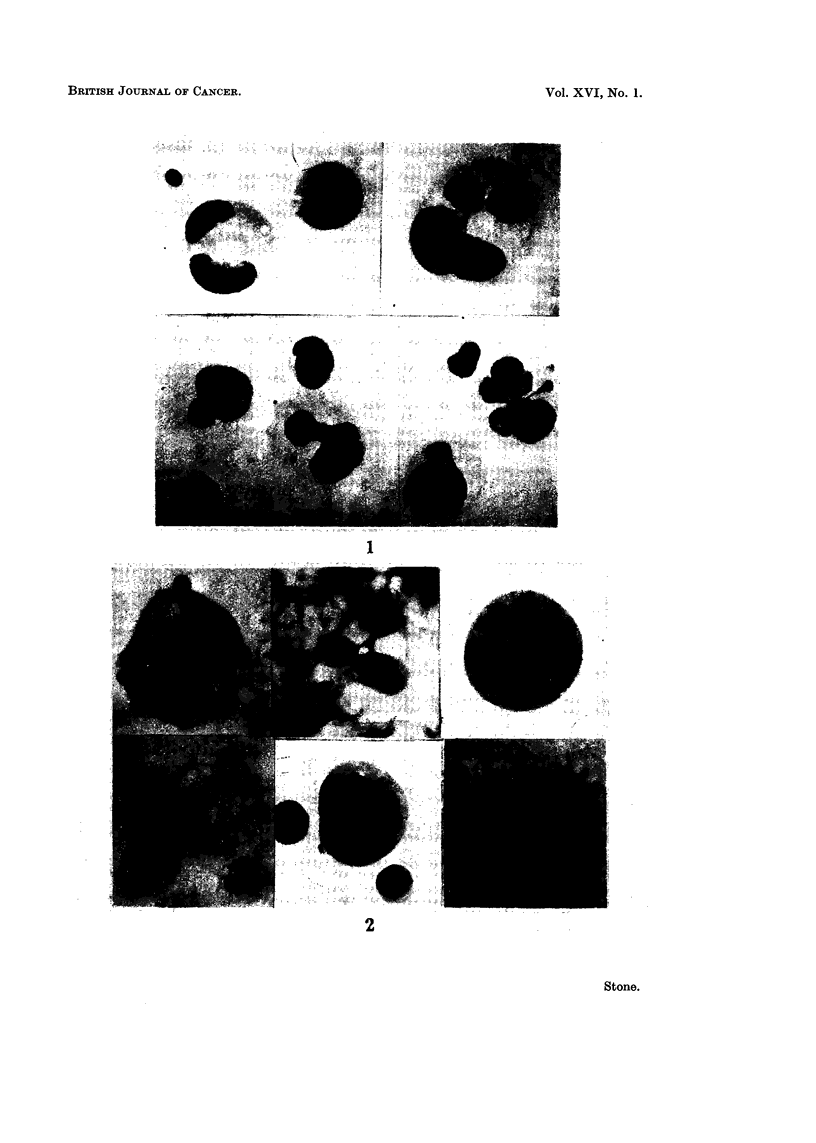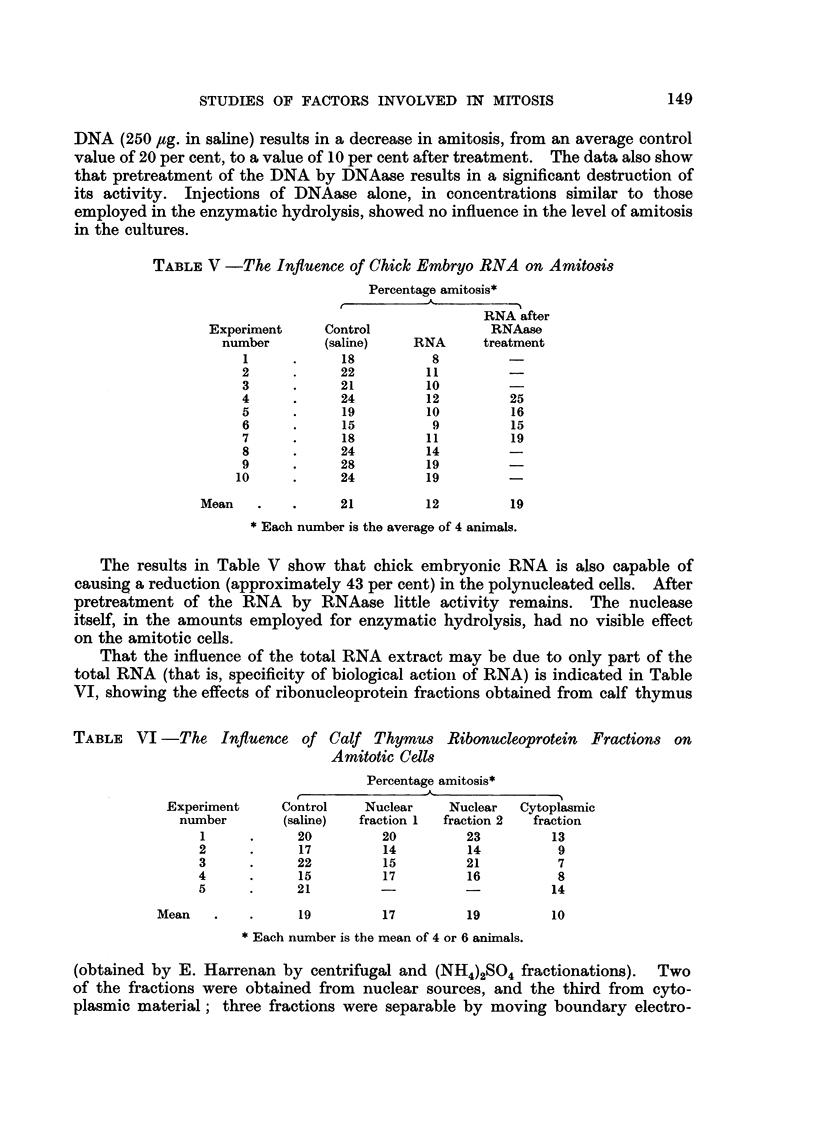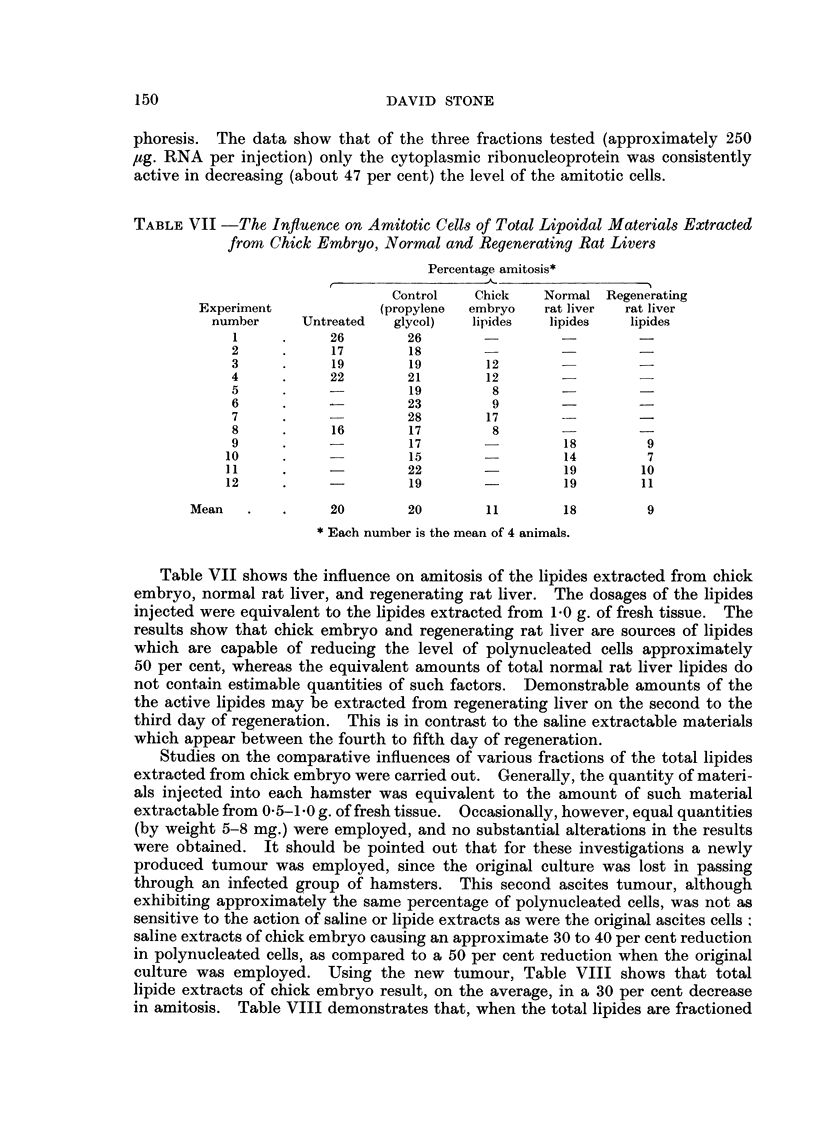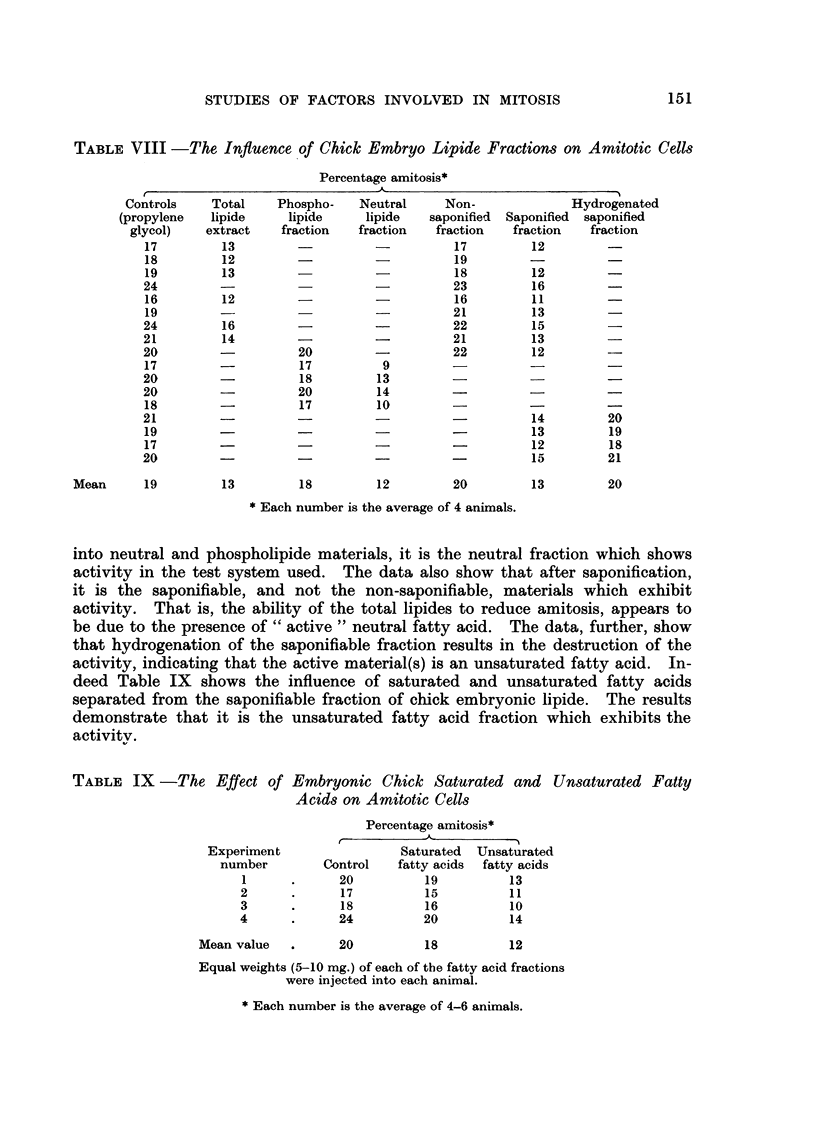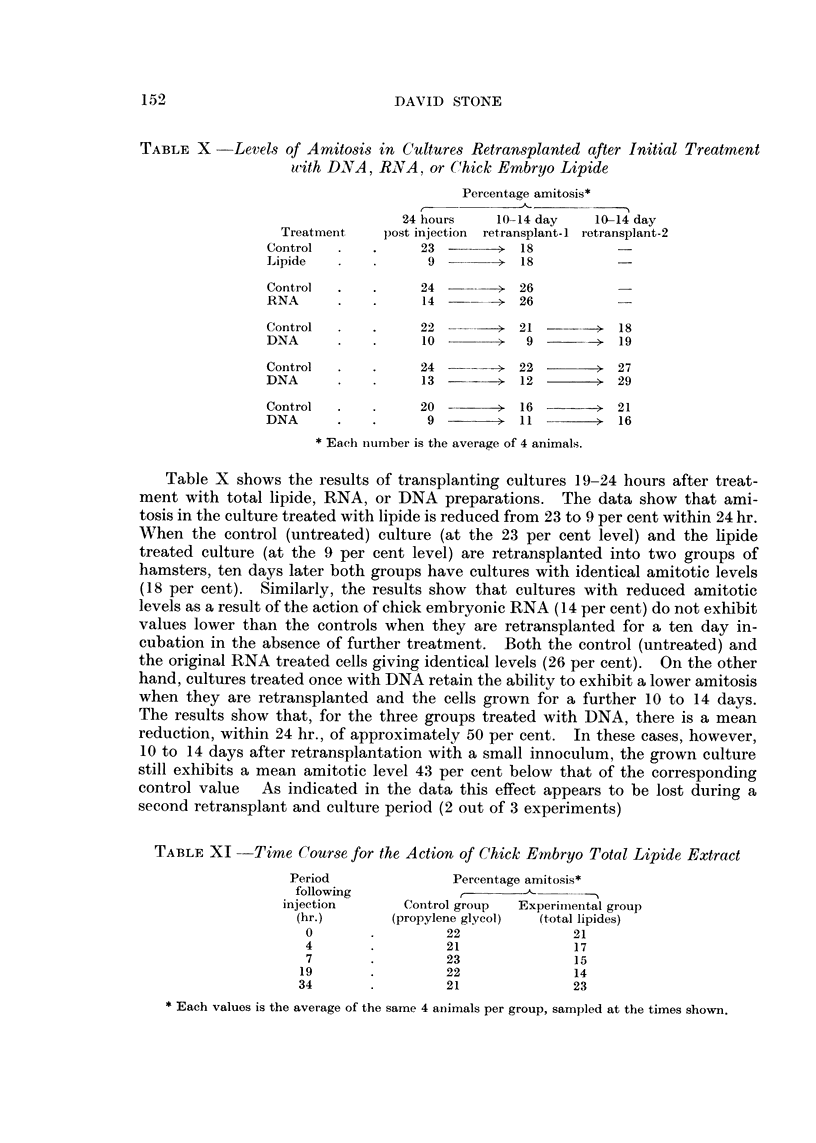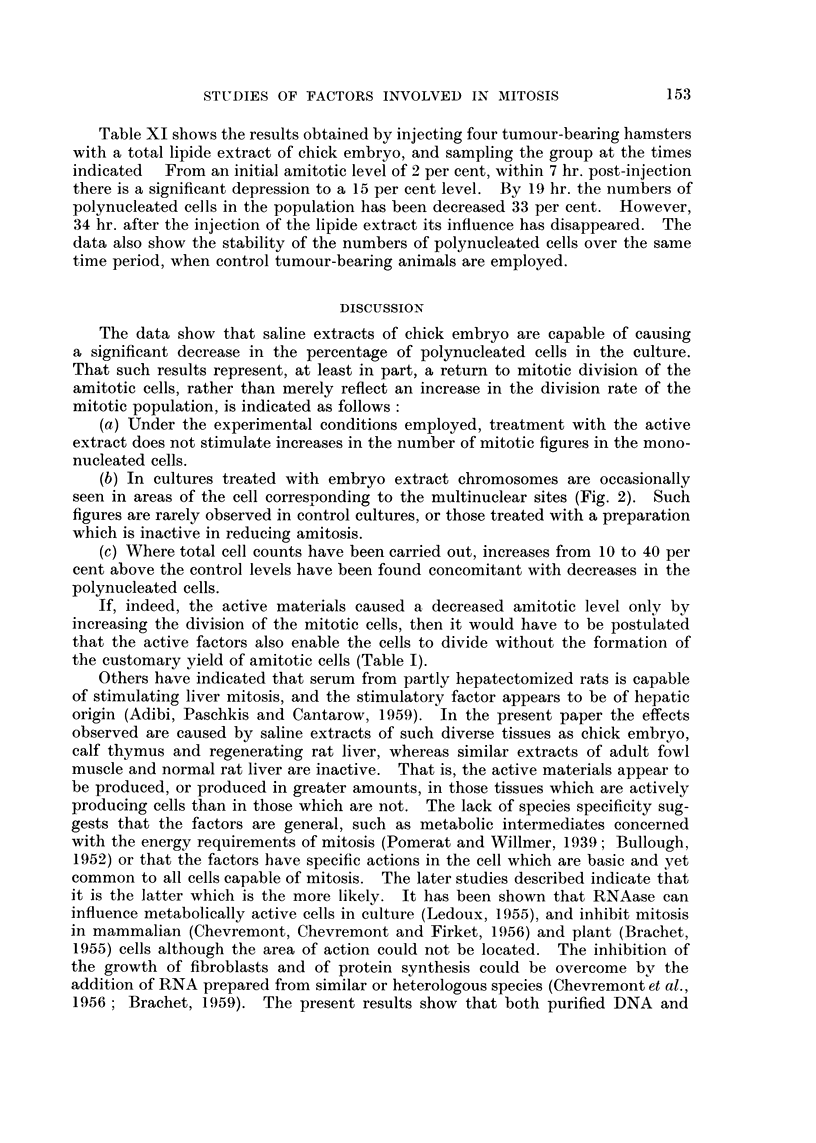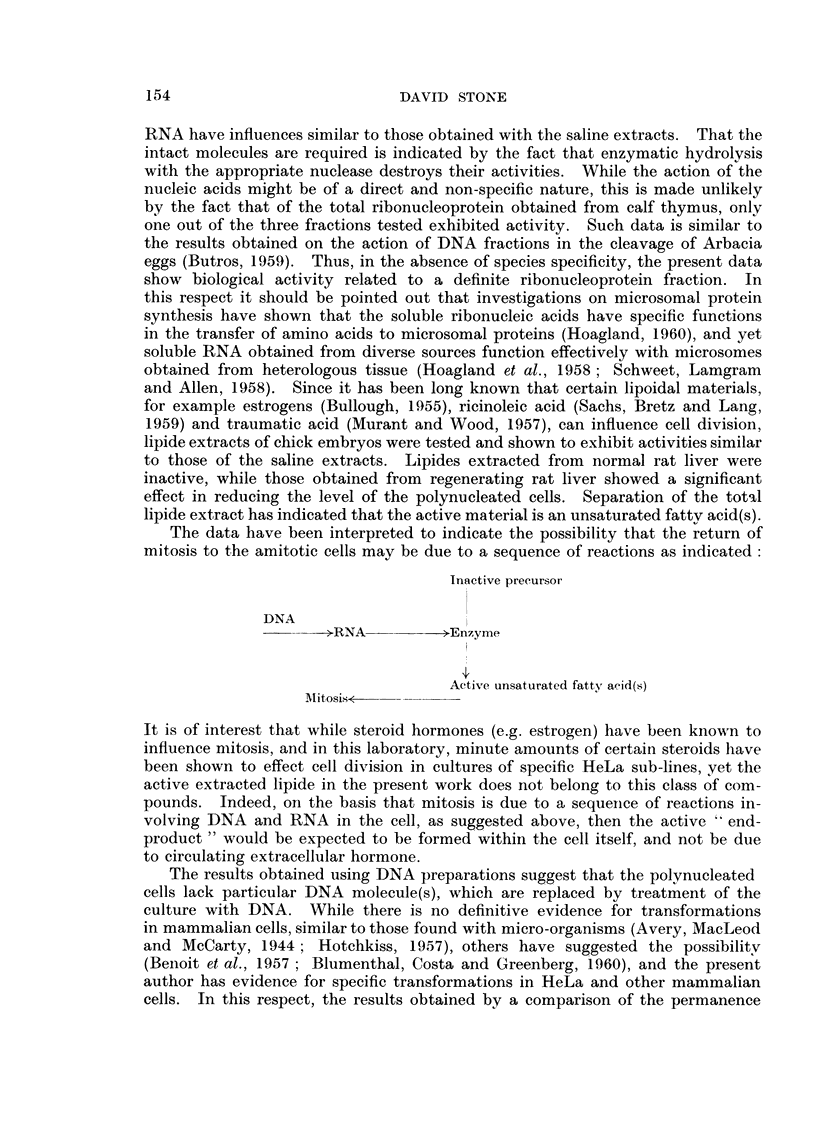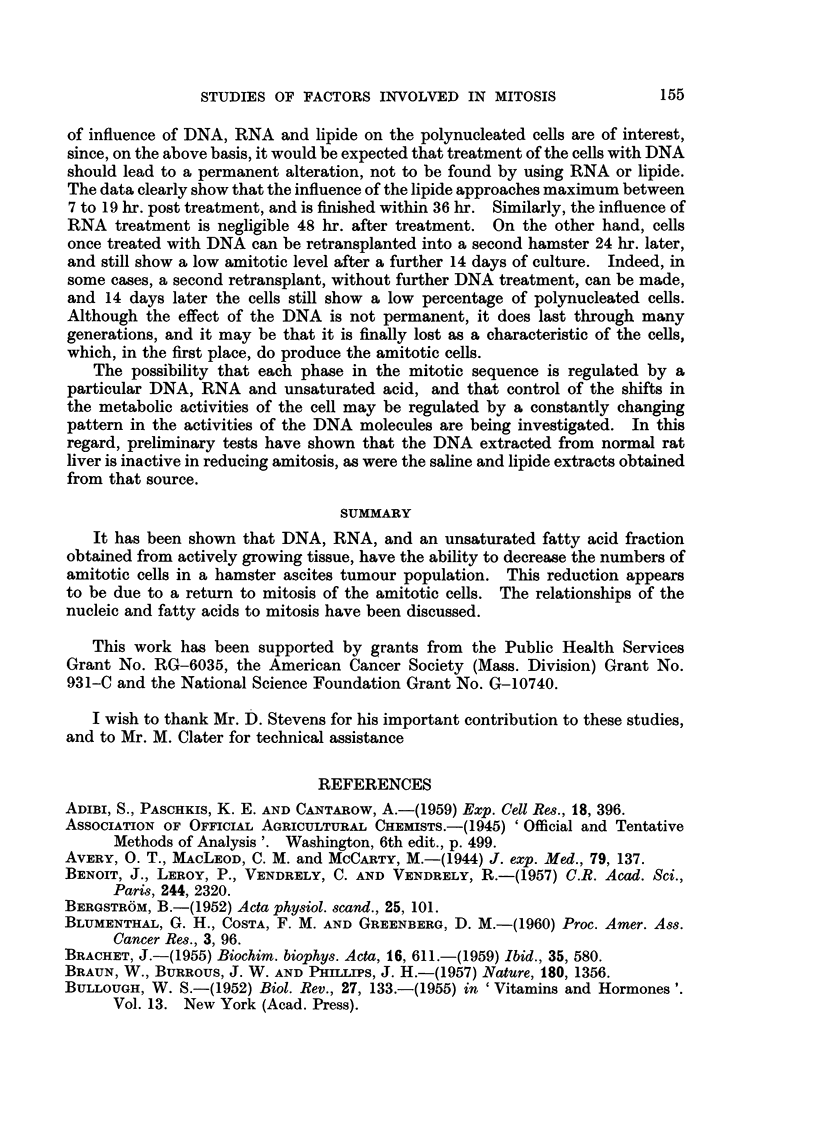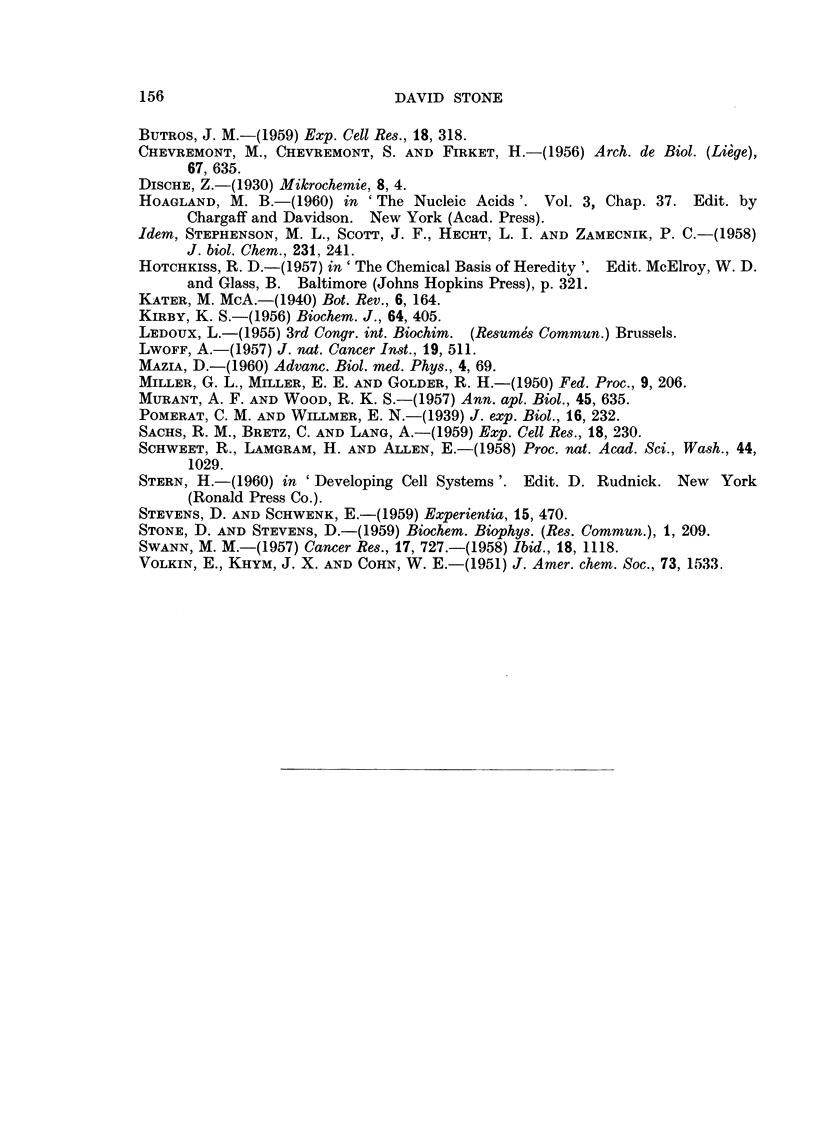# The use of Amitotic Cells for the Study of Factors Involved in Mitosis: The Possible Involvement of DNA, RNA and Unsaturated Fatty Acids

**DOI:** 10.1038/bjc.1962.15

**Published:** 1962-03

**Authors:** David Stone

## Abstract

**Images:**


### THE USE OF AMITOTIC CELLS FOR THE STUDY OF FACTORS INVOLVED IN MITOSIS: THE POSSIBLE INVOLVEMENT OF DNA, RNA AND UNSATURATED FATTY ACIDS

DAVID STONE

*From the Worcester Foundation for Experimental Biology, Shrewsbury, Massachusetts, U.S.A.*

Received for publication December 12, 1961

In recent years, investigators have become concerned with the biochemical events that take place before and during the process of mitosis (Swann, 1957, 1958; Stern, 1960; Mazia, 1960). The dependence of protein synthesis to RNA has now been clearly indicated (Hoagland, 1960) and a working hypothesis for the regulation of cellular metabolism has been put forward (Lwoff, 1957). According to such views, a control of cellular function is exerted by a multiplication or decrease of definite RNA particles, and in consequence, a regulated or altered spectrum in the enzymatic activities of the cell.

The present paper describes some experiments carried out in an attempt to investigate the role of nucleic acids with respect to the changes in cellular activites taking place during mitosis. Cultures of a hamster ascites population obtained by others (Stevens and Schwenk, 1959), have been shown to contain a high percentage of polynucleated cells which do not appear to be actively involved in cell population increase, since extensive observations by stained preparations, and by phase-microscopy of viable preparations, have revealed no evidence of cytokinesis or mitotic figures in such cells. Examination of the polynucleated cells has shown that they are produced by the budding of a daughter nucleus from the parent nucleus (Fig. 1), a type of nuclear division which has been classified as amitotic division (Kater, 1940). As briefly described in a preliminary report (Stone and Stevens, 1959), a working hypothesis has been considered, whereby, during division of the mitotic population cells are formed which have lost, or lose, their capacity for the initiation or completion of their mitotic apparatus. Such cells have been regarded as " mutants " of their parents, offering the opportunity to investigate requirements essential for division, by influencing amitotic cells cells to return to mitotic division. The present communication shows that saline extracts obtained from various growing tissues, have the capacity to allow amitotic cells of hamster ascites population to return to mitotic division; further, DNA and RNA obtained from such active tissues have similar influences. The results indicate some specificity to the action of RNA, since only a particular fraction of the total RNA is active in the test system employed. An active unsaturated fatty acid fraction has also been obtained. The possible relationships between DNA, RNA and the lipide to mitosis are discussed.

### METHODS

*Culture and counting.*—Hamster ascites cells obtained from a solid tumour (Stevens and Schwenk, 1959) were cultured for 10–14 days in the peritoneal cavities of adult female golden hamster. The animals with developed tumours

(10–20 ml. by volume) were then divided into groups of four to six to receive intraperitoneal injections of the material under test, or of control solution. Unless otherwise stated, 19 to 24 hours later ascites fluid was removed from each animal and samples taken for smear and Feulgen staining, in order to determine the percentage of amitotic cells in the total tumour population. Usually duplicate slides were made, and 1000 cells were counted on each slide. Occasionally, total tumour cell counts were made using a standard hemocytometer. The tumour was carried by injecting new hamsters with 0·5 ml. of the ascites fluid from the untreated tumour bearing group.

*Saline extracts.*—Tissue was weighed, minced in a known quantity of isotonic saline (1 g. wet weight tissue to 4 ml. saline), and homogenized in a loose-fitting glass homogenizer. The homogenate was decanted and centrifuged at 3–5000 r.p.m. for 10 to 15 minutes to remove cell debris. The resulting supernate (tissue extract) was stored by freezing until required for injection. All manipulations in the preparation of the saline extracts were carried out in the cold, using as aseptic a technique as possible. Chick embryos used were 11–13 days old.

*Nucleic acid prearations.*—DNA from cell homogenates was obtained and deproteinized by a method similar to that employed in the preparation of DNA-transforming-factors from micro-organisms (Braun, Burrous and Phillips, 1957). RNA was extracted by procedures similar to those used to obtain soluble RNA (Kirby, 1956). Purity of the preparations was established by U.V. spectrophotometry, and by a comparison of these values to the ribose (Miller, Miller and Golder, 1950) ; and deoxyribose (Dische, 1930) contents, using yeast RNA and sperm DNA, respectively, as standard. Enzymatic hydrolyses of the DNA (Volkin, Khym and Cohn, 1951), and the RNA preparations were carried out using nucleases obtained from the Worthington Biochemical Corporation.

*Lipide extracts.*—Total lipoidal materials were obtained by homogenization of the tissue in acetone (3 times), followed by a similar extraction of the dehydrated tissue with ethyl acetate (3 times). The combined extracts were taken to near dryness *in vacuo*, and partitioned between water and ethyl acetate. The water-washed ethyl acetate extract was dried over anhydrous sodium sulfate and then taken to dryness. Saponification of total lipide was accomplished in 75 per cent ethanol containing 7 per cent KOH, by refluxing for 6 to 7 hours followed by standing at room temperature overnight. After addition of water and extraction of the non-saponifiable materials with pentane (3 times), the mixture was acidified to Congo Red paper, and the saponifiable materials extracted with pentane (3 times). The pentane extracts were dried *in vacuo*.

Isolation of the phospholipides from the total lipide extract was accomplished by the $MgCl_2$ precipitation method (Bergström, 1952). Hydrogenation of the saponified materials was carried out in ethyl acetate using platinum black catalyst. Separation of the saturated and unsaturated fatty acids was carried out by the Twitchell lead precipitation method (Association of Optical Agricultural Chemists, 1945). Usually the extraction of one embryo weighing between 12–14 g. (wet weight) resulted in approximately 60–100 mg. total lipides. Of this, 30–50 mg. were saponifiables, 40 per cent remained in the non-saponifiable fraction, and about 10 per cent of the total lipides were phospholipides. Approximately 60 per cent, by weight of the saponifiables were found to be unsaturated.

All the lipides tested were injected in suspension in propylene glycol.

### RESULTS

Table I shows the relative constancy in the percentage production of polynucleated cells in the growing tumour, when samples are taken daily (8th to 14h day) from a group of four tumour-bearing hamsters.

TABLE I —*Constancy of the Culture Population*

| | Days of culture | | | | | | |
|---|---|---|---|---|---|---|---|
| | 8 | 9 | 10 | 11 | 12 | 13 | 14 |
| Percentage of amitotic cells* . | 22 | 24 | 24 | 25 | 22 | 25 | 24 |

* Each number is the average of 4 animals.

TABLE II —*Influence of Saline Extracts of Chick Embryo on Amitotic Cells*

| | Percentage amitotic cells* | | | |
|---|---|---|---|---|
| Experiment number | Untreated controls | Saline-injected controls | Embryo extract | Boiled embryo extract |
| 1 . | 25 | 26 | 16 | — |
| 2 . | — | 22 | 13 | — |
| 3 . | 22 | 22 | 9 | 20 |
| 4 . | 22 | 22 | 19 | — |
| 5 . | — | 22 | 12 | — |
| 6 . | 24 | 23 | 7 | 21 |
| 7 . | 22 | 21 | 11 | — |
| 8 . | — | 21 | 12 | — |
| 9 . | — | 18 | 9 | — |
| 10 . | 18 | 18 | 10 | 17 |
| 11 . | 15 | 16 | 9 | 14 |
| Mean value . | 21 | 22 | 12 | 18 |

* Each number is the average of 4 animals.

Table II shows the percentage of amitotic cells 19 to 24 hours after the injection of saline (control) and saline extracts (experimental) of chick embryo equivalent to approximately 0·5 g. wet weight embryonic tissue. The data demonstrate that while saline alone and boiled embryo extract have little or no influence, the unboiled embryonic extract always results in a significant diminution in the numbers of polynucleated cells, with an average 45 per cent decrease. It should be pointed out, that, as indicated in the data, while the level of amitosis can vary considerably in passing the culture, there is generally much less variation between animals within any one group.

Saline extracts of equivalent tissue weights of calf thymus and regenerating rat liver also exhibit this activity, while similar extracts of adult fowl muscle and normal rat liver are inactive, as may be seen in Table III.

Generally, active saline extracts can be obtained between the fourth to fifth day of liver regeneration, and by the seventh day such active factors cannot now be detected. Regardless of whether saline alone or saline tissue extracts are injected, under the experimental conditions used, the mitotic rate of the mononucleated cells (as indicated by counting mitotic figures) remains the same, at about 2 per cent. However, the appearance of chromosomes in the polynucleated cells (that is, of chromosomal patterns in several areas of the cell) is

TABLE III—*Influence of Saline Extracts of Calf Thymus, Adult Fowl Muscle, Normal Rat Liver, and Regenerating Rat Liver on Amitotic Cells*

| | Percentage amitosis* | | | | |
|---|---|---|---|---|---|
| Experiment number | Control (saline) | Calf thymus extract | Fowl muscle extract | Normal rat liver extract | Regenerating rat liver extract |
| 1 . | 18 | 11 | — | — | — |
| 2 . | 17 | 12 | — | — | — |
| 3 . | 20 | 11 | — | — | — |
| 4 . | 22 | 14 | — | — | — |
| 5 . | 23 | — | 22 | — | — |
| 6 . | 18 | — | 17 | — | — |
| 7 . | 21 | — | 19 | — | — |
| 8 . | 18 | — | — | 18 | 10 |
| 9 . | 15 | — | — | 16 | 9 |
| 10 . | 22 | — | — | 22 | — |
| 11 . | 19 | — | — | 17 | 12 |
| 12 . | 21 | — | — | 19 | 11 |
| Mean . . | 19 | 12 | 19 | 18 | 11 |

* Each number is the average of 4 animals.

rarely, if at all, observed (Fig. 1) unless the cultures have been treated with a tissue extract active in reducing amitosis. In the latter event, such chromosomal patterns, as represented in Fig. 2 may occasionally be observed (one or two per 1000 total cell count).

The results in Table IV show the effects of DNA preparations obtained from chick embryo. The data demonstrate that treatment of the tumour cells with

TABLE IV—*Influence of Chick Embryo DNA on Amitotic Cells*

| | Percentage amitosis* | | |
|---|---|---|---|
| Experiment number | Control (saline) | DNA | DNA after DNAase treatment |
| 1 . | 22 | 24† | — |
| 2 . | 21 | 20† | — |
| 3 . | 18 | 9 | — |
| 4 . | 24 | 7 | 21 |
| 5 . | 14 | 8 | — |
| 6 . | 16 | 10 | 15 |
| 7 . | 19 | 9 | 17 |
| 8 . | 24 | 13 | — |
| 9 . | 19 | 7 | 16 |
| 10 . | 21 | 16 | — |
| 11 . | 22 | 10 | — |
| Mean . . | 20 | 10 | 17 |

* Each number is the average of 4 animals.
† U.V. showed that these preparations did not contain nucleic acids.

---

### EXPLANATION OF PLATE.

FIG. 1.—This figure shows typical multinucleated cells, and several stage of nuclear budding.
FIG. 2.—This is representative of the chromosomal areas formed in the presence of factors which reduce the numbers of multinucleated cells in the cell population.

1

2

DNA (250 $\mu g$. in saline) results in a decrease in amitosis, from an average control value of 20 per cent, to a value of 10 per cent after treatment. The data also show that pretreatment of the DNA by DNAase results in a significant destruction of its activity. Injections of DNAase alone, in concentrations similar to those employed in the enzymatic hydrolysis, showed no influence in the level of amitosis in the cultures.

TABLE V—*The Influence of Chick Embryo RNA on Amitosis*

| Experiment number | Percentage amitosis* Control (saline) | RNA | RNA after RNAase treatment |
|---|---|---|---|
| 1 | 18 | 8 | — |
| 2 | 22 | 11 | — |
| 3 | 21 | 10 | — |
| 4 | 24 | 12 | 25 |
| 5 | 19 | 10 | 16 |
| 6 | 15 | 9 | 15 |
| 7 | 18 | 11 | 19 |
| 8 | 24 | 14 | — |
| 9 | 28 | 19 | — |
| 10 | 24 | 19 | — |
| Mean | 21 | 12 | 19 |

* Each number is the average of 4 animals.

The results in Table V show that chick embryonic RNA is also capable of causing a reduction (approximately 43 per cent) in the polynucleated cells. After pretreatment of the RNA by RNAase little activity remains. The nuclease itself, in the amounts employed for enzymatic hydrolysis, had no visible effect on the amitotic cells.

That the influence of the total RNA extract may be due to only part of the total RNA (that is, specificity of biological action of RNA) is indicated in Table VI, showing the effects of ribonucleoprotein fractions obtained from calf thymus

TABLE VI—*The Influence of Calf Thymus Ribonucleoprotein Fractions on Amitotic Cells*

| Experiment number | Percentage amitosis* Control (saline) | Nuclear fraction 1 | Nuclear fraction 2 | Cytoplasmic fraction |
|---|---|---|---|---|
| 1 | 20 | 20 | 23 | 13 |
| 2 | 17 | 14 | 14 | 9 |
| 3 | 22 | 15 | 21 | 7 |
| 4 | 15 | 17 | 16 | 8 |
| 5 | 21 | — | — | 14 |
| Mean | 19 | 17 | 19 | 10 |

* Each number is the mean of 4 or 6 animals.

(obtained by E. Harrenan by centrifugal and $(NH_4)_2SO_4$ fractionations). Two of the fractions were obtained from nuclear sources, and the third from cytoplasmic material; three fractions were separable by moving boundary electrophoresis. The data show that of the three fractions tested (approximately 250 $\mu$g. RNA per injection) only the cytoplasmic ribonucleoprotein was consistently active in decreasing (about 47 per cent) the level of the amitotic cells.

TABLE VII —*The Influence on Amitotic Cells of Total Lipoidal Materials Extracted from Chick Embryo, Normal and Regenerating Rat Livers*

| Experiment number | Percentage amitosis* Untreated | Control (propylene glycol) | Chick embryo lipides | Normal rat liver lipides | Regenerating rat liver lipides |
|---|---|---|---|---|---|
| 1 | 26 | 26 | — | — | — |
| 2 | 17 | 18 | — | — | — |
| 3 | 19 | 19 | 12 | — | — |
| 4 | 22 | 21 | 12 | — | — |
| 5 | — | 19 | 8 | — | — |
| 6 | — | 23 | 9 | — | — |
| 7 | — | 28 | 17 | — | — |
| 8 | 16 | 17 | 8 | — | — |
| 9 | — | 17 | — | 18 | 9 |
| 10 | — | 15 | — | 14 | 7 |
| 11 | — | 22 | — | 19 | 10 |
| 12 | — | 19 | — | 19 | 11 |
| Mean | 20 | 20 | 11 | 18 | 9 |

* Each number is the mean of 4 animals.

Table VII shows the influence on amitosis of the lipides extracted from chick embryo, normal rat liver, and regenerating rat liver. The dosages of the lipides injected were equivalent to the lipides extracted from 1·0 g. of fresh tissue. The results show that chick embryo and regenerating rat liver are sources of lipides which are capable of reducing the level of polynucleated cells approximately 50 per cent, whereas the equivalent amounts of total normal rat liver lipides do not contain estimable quantities of such factors. Demonstrable amounts of the the active lipides may be extracted from regenerating liver on the second to the third day of regeneration. This is in contrast to the saline extractable materials which appear between the fourth to fifth day of regeneration.

Studies on the comparative influences of various fractions of the total lipides extracted from chick embryo were carried out. Generally, the quantity of materials injected into each hamster was equivalent to the amount of such material extractable from 0·5–1·0 g. of fresh tissue. Occasionally, however, equal quantities (by weight 5–8 mg.) were employed, and no substantial alterations in the results were obtained. It should be pointed out that for these investigations a newly produced tumour was employed, since the original culture was lost in passing through an infected group of hamsters. This second ascites tumour, although exhibiting approximately the same percentage of polynucleated cells, was not as sensitive to the action of saline or lipide extracts as were the original ascites cells ; saline extracts of chick embryo causing an approximate 30 to 40 per cent reduction in polynucleated cells, as compared to a 50 per cent reduction when the original culture was employed. Using the new tumour, Table VIII shows that total lipide extracts of chick embryo result, on the average, in a 30 per cent decrease in amitosis. Table VIII demonstrates that, when the total lipides are fractioned

TABLE VIII —*The Influence of Chick Embryo Lipide Fractions on Amitotic Cells*

| | Percentage amitosis* | | | | | | |
|---|---|---|---|---|---|---|---|
| | Controls (propylene glycol) | Total lipide extract | Phospho- lipide fraction | Neutral lipide fraction | Non- saponified fraction | Saponified fraction | Hydrogenated saponified fraction |
| | 17 | 13 | — | — | 17 | 12 | — |
| | 18 | 12 | — | — | 19 | — | — |
| | 19 | 13 | — | — | 18 | 12 | — |
| | 24 | — | — | — | 23 | 16 | — |
| | 16 | 12 | — | — | 16 | 11 | — |
| | 19 | — | — | — | 21 | 13 | — |
| | 24 | 16 | — | — | 22 | 15 | — |
| | 21 | 14 | — | — | 21 | 13 | — |
| | 20 | — | 20 | — | 22 | 12 | — |
| | 17 | — | 17 | 9 | — | — | — |
| | 20 | — | 18 | 13 | — | — | — |
| | 20 | — | 20 | 14 | — | — | — |
| | 18 | — | 17 | 10 | — | — | — |
| | 21 | — | — | — | — | 14 | 20 |
| | 19 | — | — | — | — | 13 | 19 |
| | 17 | — | — | — | — | 12 | 18 |
| | 20 | — | — | — | — | 15 | 21 |
| Mean | 19 | 13 | 18 | 12 | 20 | 13 | 20 |

* Each number is the average of 4 animals.

into neutral and phospholipide materials, it is the neutral fraction which shows activity in the test system used. The data also show that after saponification, it is the saponifiable, and not the non-saponifiable, materials which exhibit activity. That is, the ability of the total lipides to reduce amitosis, appears to be due to the presence of " active " neutral fatty acid. The data, further, show that hydrogenation of the saponifiable fraction results in the destruction of the activity, indicating that the active material(s) is an unsaturated fatty acid. Indeed Table IX shows the influence of saturated and unsaturated fatty acids separated from the saponifiable fraction of chick embryonic lipide. The results demonstrate that it is the unsaturated fatty acid fraction which exhibits the activity.

TABLE IX —*The Effect of Embryonic Chick Saturated and Unsaturated Fatty Acids on Amitotic Cells*

| | Percentage amitosis* | | |
|---|---|---|---|
| Experiment number | Control | Saturated fatty acids | Unsaturated fatty acids |
| 1 . | 20 | 19 | 13 |
| 2 . | 17 | 15 | 11 |
| 3 . | 18 | 16 | 10 |
| 4 . | 24 | 20 | 14 |
| Mean value . | 20 | 18 | 12 |

Equal weights (5–10 mg.) of each of the fatty acid fractions were injected into each animal.

* Each number is the average of 4–6 animals.

TABLE X —*Levels of Amitosis in Cultures Retransplanted after Initial Treatment with DNA, RNA, or Chick Embryo Lipide*

| Treatment | Percentage amitosis* 24 hours post injection | | 10–14 day retransplant-1 | | 10–14 day retransplant-2 |
|---|---|---|---|---|---|
| Control . . | 23 | → | 18 | | — |
| Lipide . . | 9 | → | 18 | | — |
| Control . . | 24 | → | 26 | | — |
| RNA . . | 14 | → | 26 | | — |
| Control . . | 22 | → | 21 | → | 18 |
| DNA . . | 10 | → | 9 | → | 19 |
| Control . . | 24 | → | 22 | → | 27 |
| DNA . . | 13 | → | 12 | → | 29 |
| Control . . | 20 | → | 16 | → | 21 |
| DNA . . | 9 | → | 11 | → | 16 |

* Each number is the average of 4 animals.

Table X shows the results of transplanting cultures 19–24 hours after treatment with total lipide, RNA, or DNA preparations. The data show that amitosis in the culture treated with lipide is reduced from 23 to 9 per cent within 24 hr. When the control (untreated) culture (at the 23 per cent level) and the lipide treated culture (at the 9 per cent level) are retransplanted into two groups of hamsters, ten days later both groups have cultures with identical amitotic levels (18 per cent). Similarly, the results show that cultures with reduced amitotic levels as a result of the action of chick embryonic RNA (14 per cent) do not exhibit values lower than the controls when they are retransplanted for a ten day incubation in the absence of further treatment. Both the control (untreated) and the original RNA treated cells giving identical levels (26 per cent). On the other hand, cultures treated once with DNA retain the ability to exhibit a lower amitosis when they are retransplanted and the cells grown for a further 10 to 14 days. The results show that, for the three groups treated with DNA, there is a mean reduction, within 24 hr., of approximately 50 per cent. In these cases, however, 10 to 14 days after retransplantation with a small innoculum, the grown culture still exhibits a mean amitotic level 43 per cent below that of the corresponding control value As indicated in the data this effect appears to be lost during a second retransplant and culture period (2 out of 3 experiments)

TABLE XI —*Time Course for the Action of Chick Embryo Total Lipide Extract*

| Period following injection (hr.) | Percentage amitosis* Control group (propylene glycol) | Experimental group (total lipides) |
|---|---|---|
| 0 . | 22 | 21 |
| 4 . | 21 | 17 |
| 7 . | 23 | 15 |
| 19 . | 22 | 14 |
| 34 . | 21 | 23 |

* Each values is the average of the same 4 animals per group, sampled at the times shown.

Table XI shows the results obtained by injecting four tumour-bearing hamsters with a total lipide extract of chick embryo, and sampling the group at the times indicated From an initial amitotic level of 2 per cent, within 7 hr. post-injection there is a significant depression to a 15 per cent level. By 19 hr. the numbers of polynucleated cells in the population has been decreased 33 per cent. However, 34 hr. after the injection of the lipide extract its influence has disappeared. The data also show the stability of the numbers of polynucleated cells over the same time period, when control tumour-bearing animals are employed.

### DISCUSSION

The data show that saline extracts of chick embryo are capable of causing a significant decrease in the percentage of polynucleated cells in the culture. That such results represent, at least in part, a return to mitotic division of the amitotic cells, rather than merely reflect an increase in the division rate of the mitotic population, is indicated as follows :

(*a*) Under the experimental conditions employed, treatment with the active extract does not stimulate increases in the number of mitotic figures in the mononucleated cells.

(*b*) In cultures treated with embryo extract chromosomes are occasionally seen in areas of the cell corresponding to the multinuclear sites (Fig. 2). Such figures are rarely observed in control cultures, or those treated with a preparation which is inactive in reducing amitosis.

(*c*) Where total cell counts have been carried out, increases from 10 to 40 per cent above the control levels have been found concomitant with decreases in the polynucleated cells.

If, indeed, the active materials caused a decreased amitotic level only by increasing the division of the mitotic cells, then it would have to be postulated that the active factors also enable the cells to divide without the formation of the customary yield of amitotic cells (Table I).

Others have indicated that serum from partly hepatectomized rats is capable of stimulating liver mitosis, and the stimulatory factor appears to be of hepatic origin (Adibi, Paschkis and Cantarow, 1959). In the present paper the effects observed are caused by saline extracts of such diverse tissues as chick embryo, calf thymus and regenerating rat liver, whereas similar extracts of adult fowl muscle and normal rat liver are inactive. That is, the active materials appear to be produced, or produced in greater amounts, in those tissues which are actively producing cells than in those which are not. The lack of species specificity suggests that the factors are general, such as metabolic intermediates concerned with the energy requirements of mitosis (Pomerat and Willmer, 1939 ; Bullough, 1952) or that the factors have specific actions in the cell which are basic and yet common to all cells capable of mitosis. The later studies described indicate that it is the latter which is the more likely. It has been shown that RNAase can influence metabolically active cells in culture (Ledoux, 1955), and inhibit mitosis in mammalian (Chevremont, Chevremont and Firket, 1956) and plant (Brachet, 1955) cells although the area of action could not be located. The inhibition of the growth of fibroblasts and of protein synthesis could be overcome by the addition of RNA prepared from similar or heterologous species (Chevremont *et al.*, 1956 ; Brachet, 1959). The present results show that both purified DNA and

RNA have influences similar to those obtained with the saline extracts. That the intact molecules are required is indicated by the fact that enzymatic hydrolysis with the appropriate nuclease destroys their activities. While the action of the nucleic acids might be of a direct and non-specific nature, this is made unlikely by the fact that of the total ribonucleoprotein obtained from calf thymus, only one out of the three fractions tested exhibited activity. Such data is similar to the results obtained on the action of DNA fractions in the cleavage of Arbacia eggs (Butros, 1959). Thus, in the absence of species specificity, the present data show biological activity related to a definite ribonucleoprotein fraction. In this respect it should be pointed out that investigations on microsomal protein synthesis have shown that the soluble ribonucleic acids have specific functions in the transfer of amino acids to microsomal proteins (Hoagland, 1960), and yet soluble RNA obtained from diverse sources function effectively with microsomes obtained from heterologous tissue (Hoagland *et al.*, 1958 ; Schweet, Lamgram and Allen, 1958). Since it has been long known that certain lipoidal materials, for example estrogens (Bullough, 1955), ricinoleic acid (Sachs, Bretz and Lang, 1959) and traumatic acid (Murant and Wood, 1957), can influence cell division, lipide extracts of chick embryos were tested and shown to exhibit activities similar to those of the saline extracts. Lipides extracted from normal rat liver were inactive, while those obtained from regenerating rat liver showed a significant effect in reducing the level of the polynucleated cells. Separation of the total lipide extract has indicated that the active material is an unsaturated fatty acid(s).

The data have been interpreted to indicate the possibility that the return of mitosis to the amitotic cells may be due to a sequence of reactions as indicated :

It is of interest that while steroid hormones (e.g. estrogen) have been known to influence mitosis, and in this laboratory, minute amounts of certain steroids have been shown to effect cell division in cultures of specific HeLa sub-lines, yet the active extracted lipide in the present work does not belong to this class of compounds. Indeed, on the basis that mitosis is due to a sequence of reactions involving DNA and RNA in the cell, as suggested above, then the active " end-product " would be expected to be formed within the cell itself, and not be due to circulating extracellular hormone.

The results obtained using DNA preparations suggest that the polynucleated cells lack particular DNA molecule(s), which are replaced by treatment of the culture with DNA. While there is no definitive evidence for transformations in mammalian cells, similar to those found with micro-organisms (Avery, MacLeod and McCarty, 1944 ; Hotchkiss, 1957), others have suggested the possibility (Benoit *et al.*, 1957 ; Blumenthal, Costa and Greenberg, 1960), and the present author has evidence for specific transformations in HeLa and other mammalian cells. In this respect, the results obtained by a comparison of the permanence of influence of DNA, RNA and lipide on the polynucleated cells are of interest, since, on the above basis, it would be expected that treatment of the cells with DNA should lead to a permanent alteration, not to be found by using RNA or lipide. The data clearly show that the influence of the lipide approaches maximum between 7 to 19 hr. post treatment, and is finished within 36 hr. Similarly, the influence of RNA treatment is negligible 48 hr. after treatment. On the other hand, cells once treated with DNA can be retransplanted into a second hamster 24 hr. later, and still show a low amitotic level after a further 14 days of culture. Indeed, in some cases, a second retransplant, without further DNA treatment, can be made, and 14 days later the cells still show a low percentage of polynucleated cells. Although the effect of the DNA is not permanent, it does last through many generations, and it may be that it is finally lost as a characteristic of the cells, which, in the first place, do produce the amitotic cells.

The possibility that each phase in the mitotic sequence is regulated by a particular DNA, RNA and unsaturated acid, and that control of the shifts in the metabolic activities of the cell may be regulated by a constantly changing pattern in the activities of the DNA molecules are being investigated. In this regard, preliminary tests have shown that the DNA extracted from normal rat liver is inactive in reducing amitosis, as were the saline and lipide extracts obtained from that source.

SUMMARY

It has been shown that DNA, RNA, and an unsaturated fatty acid fraction obtained from actively growing tissue, have the ability to decrease the numbers of amitotic cells in a hamster ascites tumour population. This reduction appears to be due to a return to mitosis of the amitotic cells. The relationships of the nucleic and fatty acids to mitosis have been discussed.

This work has been supported by grants from the Public Health Services Grant No. RG–6035, the American Cancer Society (Mass. Division) Grant No. 931–C and the National Science Foundation Grant No. G–10740.

I wish to thank Mr. D. Stevens for his important contribution to these studies, and to Mr. M. Clater for technical assistance

### REFERENCES

Adibi, S., Paschkis, K. E. and Cantarow, A.—(1959) *Exp. Cell Res.*, **18**, 396.

Association of Official Agricultural Chemists.—(1945) ‘ Official and Tentative Methods of Analysis ’. Washington, 6th edit., p. 499.

Avery, O. T., MacLeod, C. M. and McCarty, M.—(1944) *J. exp. Med.*, **79**, 137.

Benoit, J., Leroy, P., Vendrely, C. and Vendrely, R.—(1957) *C.R. Acad. Sci., Paris*, **244**, 2320.

Bergström, B.—(1952) *Acta physiol. scand.*, **25**, 101.

Blumenthal, G. H., Costa, F. M. and Greenberg, D. M.—(1960) *Proc. Amer. Ass. Cancer Res.*, **3**, 96.

Brachet, J.—(1955) *Biochim. biophys. Acta*, **16**, 611.—(1959) *Ibid.*, **35**, 580.

Braun, W., Burrous, J. W. and Phillips, J. H.—(1957) *Nature*, **180**, 1356.

Bullough, W. S.—(1952) *Biol. Rev.*, **27**, 133.—(1955) *in* ‘ Vitamins and Hormones ’. Vol. 13. New York (Acad. Press).

Butros, J. M.—(1959) *Exp. Cell Res.*, **18**, 318.
Chevremont, M., Chevremont, S. and Firket, H.—(1956) *Arch. de Biol.* (*Liège*), **67**, 635.
Dische, Z.—(1930) *Mikrochemie*, **8**, 4.
Hoagland, M. B.—(1960) *in* 'The Nucleic Acids'. Vol. 3, Chap. 37. Edit. by Chargaff and Davidson. New York (Acad. Press).
*Idem*, Stephenson, M. L., Scott, J. F., Hecht, L. I. and Zamecnik, P. C.—(1958) *J. biol. Chem.*, **231**, 241.
Hotchkiss, R. D.—(1957) *in* 'The Chemical Basis of Heredity'. Edit. McElroy, W. D. and Glass, B. Baltimore (Johns Hopkins Press), p. 321.
Kater, M. McA.—(1940) *Bot. Rev.*, **6**, 164.
Kirby, K. S.—(1956) *Biochem. J.*, **64**, 405.
Ledoux, L.—(1955) *3rd Congr. int. Biochim.* (*Resumés Commun.*) Brussels.
Lwoff, A.—(1957) *J. nat. Cancer Inst.*, **19**, 511.
Mazia, D.—(1960) *Advanc. Biol. med. Phys.*, **4**, 69.
Miller, G. L., Miller, E. E. and Golder, R. H.—(1950) *Fed. Proc.*, **9**, 206.
Murant, A. F. and Wood, R. K. S.—(1957) *Ann. apl. Biol.*, **45**, 635.
Pomerat, C. M. and Willmer, E. N.—(1939) *J. exp. Biol.*, **16**, 232.
Sachs, R. M., Bretz, C. and Lang, A.—(1959) *Exp. Cell Res.*, **18**, 230.
Schweet, R., Lamgram, H. and Allen, E.—(1958) *Proc. nat. Acad. Sci., Wash.*, **44**, 1029.
Stern, H.—(1960) *in* 'Developing Cell Systems'. Edit. D. Rudnick. New York (Ronald Press Co.).
Stevens, D. and Schwenk, E.—(1959) *Experientia*, **15**, 470.
Stone, D. and Stevens, D.—(1959) *Biochem. Biophys.* (*Res. Commun.*), **1**, 209.
Swann, M. M.—(1957) *Cancer Res.*, **17**, 727.—(1958) *Ibid.*, **18**, 1118.
Volkin, E., Khym, J. X. and Cohn, W. E.—(1951) *J. Amer. chem. Soc.*, **73**, 1533.